# The Interaction between Arbuscular Mycorrhizal Fungi and Endophytic Bacteria Enhances Plant Growth of *Acacia gerrardii* under Salt Stress

**DOI:** 10.3389/fmicb.2016.01089

**Published:** 2016-07-19

**Authors:** Abeer Hashem, Elsayed F. Abd_Allah, Abdulaziz A. Alqarawi, Asma A. Al-Huqail, Stephan Wirth, Dilfuza Egamberdieva

**Affiliations:** ^1^Department of Botany and Microbiology, Faculty of Science, King Saud UniversityRiyadh, Saudi Arabia; ^2^Department of Mycology and Plant Disease Survey, Agriculture Research Center, Plant Pathology Research InstituteGiza, Egypt; ^3^Department of Plant Production, Faculty of Food and Agricultural Sciences, King Saud UniversityRiyadh, Saudi Arabia; ^4^Leibniz Centre for Agricultural Landscape Research, Institute of Landscape BiogeochemistryMüncheberg, Germany

**Keywords:** AMF, endophyte, *Acacia gerrardii*, salinity, nutrition

## Abstract

Microbes living symbiotically in plant tissues mutually cooperate with each other by providing nutrients for proliferation of the partner organism and have a beneficial effect on plant growth. However, few studies thus far have examined the interactive effect of endophytic bacteria and arbuscular mycorrhizal fungi (AMF) in hostile conditions and their potential to improve plant stress tolerance. In this study, we investigated how the synergistic interactions of endophytic bacteria and AMF affect plant growth, nodulation, nutrient acquisition and stress tolerance of *Acacia gerrardii* under salt stress. Plant growth varied between the treatments with both single inoculants and was higher in plants inoculated with the endophytic *B. subtilis* strain than with AMF. Co-inoculated *A. gerrardii* had a significantly greater shoot and root dry weight, nodule number, and leghemoglobin content than those inoculated with AMF or *B. subtilis* alone under salt stress. The endophytic *B*. *subtilis* could alleviate the adverse effect of salt on AMF colonization. The differences in nitrate and nitrite reductase and nitrogenase activities between uninoculated plants and those inoculated with AMF and *B. subtilis* together under stress were significant. Both inoculation treatments, either *B. subtilis* alone or combined with AMF, enhanced the N, P, K, Mg, and Ca contents and phosphatase activities in salt-stressed *A. gerrardii* tissues and reduced Na and Cl concentration, thereby protecting salt-stressed plants from ionic and osmotic stress-induced changes. In conclusion, our results indicate that endophytic bacteria and AMF contribute to a tripartite mutualistic symbiosis in *A. gerrardii* and are coordinately involved in the plant adaptation to salt stress tolerance.

## Introduction

Salinity is a devastating environmental stress factor that severely affects plant growth and development (Barnawal et al., 2014). At the global level, particularly in arid and semiarid regions, salinity is considered an important constraint, and approximately 7% of global land has a high salt concentration, making this area unavailable for agriculture (Sheng et al., 2008; Ruiz-Lozano et al., 2012). Salinity reduces plant growth through osmotic as well as ionic constraints of major physiological and biochemical processes (Ahmad, 2010; Porcel et al., 2012; Abd_Allah et al., 2015a). This may in turn alter the availability of nutrients for plant growth and affect the association with microbes living within the plant vicinity. Plants are colonized by microbes, including endophytes, nitrogen-fixing bacteria and mycorrhizal fungi, which closely cooperate with each other and can mediate important physiological processes, especially nutrient acquisition and plant tolerance to abiotic stresses (Egamberdieva, 2012; Egamberdieva et al., 2013, 2015; Berg et al., 2013; Ahanger et al., 2014a; Abd_Allah et al., 2015a). Arbuscular mycorrhiza fungi (AMF) form beneficial symbiotic associations with most plants and play a vital role in plant growth under various conditions by modifying the root system and enhancing mobilization and the uptake of several essential elements. They have also been reported to stimulate plant stress tolerance by enhancing enzymatic as well as non-enzymatic antioxidant defense systems (Wu et al., 2014; Ahmad et al., 2015), lipid peroxidation (Abd_Allah et al., 2015c), and phytohormone synthesis (Navarro et al., 2013). Endophytic bacteria colonize the internal tissues of their host plants and can promote growth, stress tolerance, and nutrient uptake and protect plants from soil-borne pathogens (Malfanova et al., 2011; Sessitsch et al., 2012). Microbes living symbiotically in plant tissues, such as mycorrhizal fungi, and nitrogen-fixing bacteria also mutually cooperate with each other by synthesizing biologically active compounds and providing nutrients for the survival and proliferation of their partner organism (Marschner et al., 2001; Egamberdieva et al., 2013). These synergies among endophytes are known to have beneficial effects on plants by improving the availability of nutrients to plants and inducing plant defense against various stresses, including drought and salinity.

The symbiotic relationship between legumes and nitrogen-fixing rhizobia is susceptible to abiotic factors, such as nutrient deficiency, salinity, drought, acidity, and soil temperature, which induce failure of the infection and nodulation processes (Slattery et al., 2001; Bouhmouch et al., 2005; Egamberdieva et al., 2013). An improvement in legume-rhizobia symbiotic performance by AMF has been reported for faba bean (*Vicia faba*) (Yinsuo et al., 2004), lucerne (*Medicago sativa*) (Ardakani et al., 2009), lentil (*Lens culinaris*) (Xavier and Germida, 2002), and common bean (*Phaseolus vulgaris*) (Tajini et al., 2012). The positive effects on plant growth and stimulation of stress tolerance by synergistic interactions of root-colonizing, plant growth-promoting bacteria (PGPR) and AMF under hostile environments have been extensively reviewed by Nadeem et al. (2014). These microbes are believed to act as essential bio-ameliorators of stress by regulating the nutritional and hormonal balance (Abd_Allah et al., 2015a,b; Egamberdieva et al., 2015, 2016) and inducing systemic tolerance to stress (Ruiz-Lozano et al., 2012).

Despite these beneficial associations of microbes, studies examining the interactions of endophytic bacteria and AMF in hostile environmental conditions are limited, especially where competition for nutrient and niches in the rhizosphere is high. This knowledge is important for our understanding of the relationship between AMF and endophytic bacteria and their potential effect on plant stress tolerance and for the development of crop management practices under hostile environmental conditions.

*Acacia gerrardii* Benth. (Talh tree) is a small leguminous shrub that is resistant to drought and salinity, forms nodules and can improve the fertility of salt-affected arid soils. This tree is widely used in Saudi Arabia for fuel, forage, medicine, food production and also for agroforestry. In this study, we hypothesize that the improved salt tolerance and growth of *A. gerrardii* are mediated by (i) the effect of endophytic bacteria on mycorrhizal development and colonization in the roots of *A. gerrardii* and (ii) nodulation, nutrient acquisition by a synergistic interaction between endophytic bacteria and AMF.

## Materials and methods

### Plants and microorganisms

#### Isolation of endophytic bacteria

Roots of Talh trees were collected from the top 20 cm of soil in a natural meadow at Khuraim in Riyadh, Saudi Arabia. The samples were wrapped in a plastic bag and brought to the laboratory, where they were incubated at 4°C until further processing. One gram of roots was surface-sterilized by immersion in 70% ethanol, followed by 5% sodium hypochlorite for 5 min, and then rinsed in sterile distilled water four to six times to eliminate the chlorine. The sterilized roots were macerated, and the extracts were placed in a tube containing 9 ml of sterile phosphate-buffered saline and then serially diluted. A 100 μl aliquot from the appropriate dilutions was plated on tryptic soy agar (TSA, Difco Laboratories, Detroit, USA) supplemented with 4% NaCl. The plates were incubated at 28°C for 3 days, and all colonies that displayed differentiable colony morphologies were selected from the plates and were re-streaked to purify the strains. To select strains with increased stress tolerance, the purified isolates were cultured in TSA medium supplemented with 3, 4, or 5% (w/v) NaCl.

#### Identification of the selected strain

A highly salt-tolerant strain, which grew well in TSA medium containing 4% NaCl, was identified. DNA was isolated by a modified version of the Töpper et al. (2010) protocol. The filters were re-suspended in 250 μl of lysozyme solution (1 mg ml^−1^ TE buffer, pH 7.4). Then, 250 μl of preheated (55°C) lysis buffer (20 μg proteinase K ml^−1^ 0.5% SDS) was added to the solution. After incubation for 30 min at 55°C, 80 μl of 5 M NaCl and 100 μl of preheated (55°C) CTAB [10% (w/v) hexadecyltrimethylammonium bromide in 0.7% NaCl] were added. The solution was incubated for a further 10 min at 65°C followed by the addition of 500 μl chloroform: isoamylalcohol (24:1, v/v). The solution was centrifuged (16,000 × g, 5 min) to separate the DNA from the remaining cell debris. Then, the top phase was transferred to a fresh tube, and the DNA was precipitated with isopropanol and later re-suspended in TE buffer (pH 7.4). Next, 16S rDNA was amplified by polymerase chain reaction (PCR) using universal forward 16SF (5′-GAGTTTGATCCTGGCTCAG-3′) and reverse 16SR (5′-GAAAGGAGGTGATCCAGCC-3′) primers (Mohanta et al., 2015). The PCR reactions were 25 μl and contained 5 μl of 5 × buffer (TaKaRa Bio Inc.), 0.5 μl of dNTP mixture (10 mM of each dNTP, TaKaRa Bio Inc.), 1 μl of 2% BSA (Promega), 0.5 μl of forward primer (10 μM), 0.5 μl of reverse primer (10 μM), 0.125 μl of One Taq DNA Polymerase (New England Biolabs), 15.375 MQ and 2 μl of template DNA. The PCR program (Bio-Rad DNA Engine) started with an initial denaturation step for 30 s at 94°C followed by 30 cycles of 15 s at 94°C, 30 s at 55°C and 1.5 min at 68°C. Before cooling to 4°C, an extension period of 20 min at 68°C was incorporated into the program. The PCR products were verified by gel electrophoresis on a 1.5% agarose gel stained with TAE (Tris-acetate-EDTA). Denaturing gradient gel electrophoresis (DGGE) was performed using the DCode system (Bio-Rad). Equal amounts of PCR products (6 μl) were loaded onto 8% acrylamide gels with a denaturing gradient of 30–55% [where 100% denaturing is defined as 7 M urea and 40% (v/v) formamide (Muyzer et al., 1995)] for optimal separation of the PCR products. DGGE gels were run for 19 h at 60 V and at 60°C in 0.59 TAE buffer and stained for 30 min with SYBR Gold (Invitrogen) diluted 10,000-fold in 19 × TAE buffer. Gels were visualized and digitized using the Fujifilm Imaging System. The PCR product was purified, and nucleotide sequences were determined using automatic LI-COR DNA Sequencer 4000 L (Lincoln, USA). The sequences were identified using the basic local alignment search tool (BLAST) and comparisons with the GenBank nucleotide data bank from the National Center for Biotechnology Information (NCBI) (http://www.ncbi.nlm.nih.gov/).

### Plant growth-promoting traits

The cellulose-degrading ability of bacterial isolates was analyzed by streaking inocula on cellulose Congo Red agar media, as described by Gupta et al. (2012). Zones of clearance around and beneath the colony were detected, indicating enzymatic degradation of cellulose.

The production of indole 3-acetic acid (IAA) was determined as described by Bano and Musarrat (2003). Briefly, bacterial strains were grown in TSB medium. After 3 days, 1 ml of each culture was pelleted by centrifugation, and the supernatant was discarded. Cell pellets were washed with 1 ml of PBS and re-suspended in PBS. One milliliter of cell suspension (corresponding to a cell density of 10^7^ cells/ml) was added to 10 ml of TSB supplemented with tryptophan (100 μg/ml). After 3 days of cultivation, 2 ml aliquots of bacterial cultures were centrifuged at 13,000 × g for 10 min. One milliliter of supernatant was transferred to a fresh tube to which 100 μg/ml of 10 mM orthophosphoric acid and 2 ml of reagent (1 ml of 0.5 M FeCl_3_ in 50 ml of 35% HClO_4_) were added. After 25 min, the absorbance of the sample was measured at 530 nm. The IAA concentration in cultures was calculated using a calibration curve of pure IAA as the standard.

The phosphate-solubilizing activity of the bacterial strains was determined on Pikovskaya agar (Pikovskaya, 1948) containing precipitated tricalcium phosphate. The bacterial culture grown in TSA medium for 2 days was streaked on the surface of Pikovskaya agar plates and incubated for 3 days. The presence of a clear zone around bacterial colonies was considered to be an indicator of positive P solubilization.

### Arbuscular mycorrhizal fungi

AMF were isolated from the soil surrounding the roots of *A. gerrardii*. AMF were extracted by wet sieving, decanting and sucrose density gradient centrifugation as described by Daniels and Skipper (1982) and modified by Utobo et al. (2011). Briefly, 100 g of soil was placed in a 10 L bucket, and 5 L of tap water was added to the soil and mixed well to produce a soil-water suspension. The suspension was left for 5 min to allow insoluble and heavy particles to settle, and the suspension was sequentially sieved through ASTM-500, ASTM-250, ASTM-150, and ASTM-50 sieves to extract the spores using a wet sieving and decanting method (Gerdemann and Nicolson, 1963). The sieved residues were filtered through Whatman filter paper No. 1. After water filtration, the filter paper was examined under a stereo-binocular microscope at 25 × magnification. Morphologically similar spores were selected for identification. AMF species were identified based on the description of subcellular structures (spore color, shape, surface ornamentation, spore contents, and wall structures) of asexual spores provided by the International Culture Collection of Vesicular and Arbuscular Mycorrhizal Fungi (INVAM 2012)^1^ and other descriptive protocols (Bethlenfalvay and Yoder, 1981; Schüßler and Walker, 2010; Redecker et al., 2013).

### Propagation of AMF in trap cultures

The trap culture protocol described by Stutz and Morton (1996) was used in the current study to propagate the most efficient mycorrhizal isolates. Sterilized sand (121°C for 3days) was inoculated with single spores from each mycorrhizal isolate. Surface-sterilized seeds [0.5% (v/v) NaOCl used] of *Sorghum sudanense* were sown (20 seeds/pot) at a depth of 2 cm in each pot (5 kg capacity) of trap cultures. The pots were incubated in a plant growth chamber at 25 ± 2°C with 18 h photoperiod, 750 μmol m^−2^ s^−1^ photosynthetic photon flux density, and 70–75% relative humidity for 3 months. Half-strength Hoagland's solution was used to irrigate the pots. The trap culture was used as the mycorrhizal inoculum and was added to the experimental soil as 25 g of trap soil culture (approx. 100 spores/g trap soil)/pot. Soil not inoculated with mycorrhiza served as the control.

### Determination of arbuscular mycorrhizal colonization

At the end of the pot experiment (12 weeks), fine roots were collected from the lateral root system and fixed in formalin/acetic acid/alcohol (v/v/v) (FAA) solution until further processing. Roots were stained with trypan blue in lactophenol (Phillips and Hayman, 1970) and assessed for mycorrhizal infection. Roots that were pigmented after clearing were bleached in alkaline hydrogen peroxide (0.5% NH_4_OH and 0.5% H_2_O_2_ v/v in water) to remove any phenolic compounds (Kormanik and McGraw, 1982) before acidification (0.05 M HCl). To assess mycorrhizal colonization, stained root segments (one cm in length) were mounted on glass slides with lactophenol and were observed under a digital computerized microscope (model DP-72, Olympus) at 20 × magnification. A minimum of 50 segments for each replicate sample were observed to assess structural colonization of AMF associated with roots. Twenty or more segments were mounted on each slide and examined under the microscope. The presence of mycelia, vesicles and arbuscules was recorded and analyzed to assess structural colonization.

### Germination of seeds

*Acacia gerrardii* seeds were surface-sterilized by immersion for 5 min in concentrated sulfuric acid followed by 3 min in 70% ethanol and were rinsed five times with sterile, distilled water.

Germination tests were carried out in Petri dishes (Ø 85 × 15 mm) containing 1% water agar. Salinity conditions were established by adding 50, 100, 150, 200, 250, and 300 mM NaCl. Twenty healthy and surface-sterilized seeds were placed on each Petri dish and were arranged in a randomized complete block design with three replications. Eventually, the Petri dishes were covered with a polyethylene sheet to avoid the loss of the moisture through evaporation and kept in the plant growth chamber at 28°C. The seeds were observed daily, and the percent germination was recorded after 10 days of incubation. Seeds were considered to have germinated when the emerging radicles were greater than 0.5 cm long.

### Plant growth condition

This experiment was carried out in the growth chamber of the Plant Production Department, Faculty of Food & Agricultural Sciences, King Saud University, Riyadh, Saudi Arabia. Talh tree seeds were provided by a personal nursery in Alghat, Riyadh that produces tree seedlings. The soil used was loamy sand soil with the following properties (%): sand (87.6), clay (7.2), silt (5.2), organic carbon (0.12), total nitrogen (0.005), pH 7.5. The sand was washed with 1.0 N H_2_SO_4_ for 1 h, followed by 1.0 N Ca carbonate, and was then washed using distilled water. The surface-sterilized seeds were sown in acid-washed sterile sand and kept in a plant growth chamber under the same conditions described for trap cultures for 1 month.

The bacterial strain was grown in TSB medium for 2 days, and 1 ml of culture suspension was pelleted by centrifugation. The supernatant was discarded, and the cell pellets were washed with 1 ml phosphate-buffered saline and diluted to a cell density of 10^8^ cells/ml. Roots of 1-month-old seedlings were immersed in the bacterial suspension for 30 min and sown in pots (1 seedling per pot) filled with 2 kg of sandy loam soil mixed with mycorrhizal inoculum [i.e., 25 g of trap soil culture or approx. 100 spores/g trap soil (*M* = 80%)/pot]. The experiment was a completely randomized design with five replicates for each treatment: (i) Control without microbes, (ii) *Bacillus subtilis* (BS), (iii) AMF, and (iv) *B. subtilis* combined with AMF (BS+AMF). Plants were grown in a greenhouse for 12 weeks with average day/night temperatures of 25°C/18°C and were supplemented with tap water as required. Salinity was established by adding NaCl to the irrigation solution to obtain a constant concentration of 250 mM. At harvest, plants were harvested carefully, and shoots were separated from roots and dried to constant weight at 100°C. Fresh nodules were used to estimate leghemoglobin (LB) levels as well as the activities of nitrite reductase, nitrate reductase, and nitrogenase. Fresh leaf samples were used to assess the content of photosynthetic pigments.

### Nodulation, leghemoglobin, and crude protein contents

The nodule fresh weight (FW) and the number of nodules per plant root were determined. The leghemoglobin concentration of root nodules was estimated using the method of Keilin and Wang (1945). Fresh nodules (2.0 g) were ground to a fine powder in liquid N_2_ and transferred to 50 mM KPO_4_ (pH 7.4) buffer containing 1 mM EDTA. The mixture was stirred until it thawed into a homogenate at a final temperature of 2°C, transferred to centrifuge tubes and centrifuged at 4°C and 10,000 × g for 10 min. The leghemoglobin-containing supernatant was collected and maintained at a known volume with 50 mM KPO_4_ (pH 7.4) buffer as described above. The color intensity that developed was recorded spectrophotometrically at 710 nm against a blank, which contained 50 mM KPO_4_ (pH 7.4) buffer with 1 mM EDTA. Leghemoglobin concentration was expressed as mg/g nodule (FW). The micro-Kjeldahl method described by Allen (1953) was used to estimate the total nitrogen content of oven-dried (110°C for two successive weights) nodules. Crude protein concentration (%, w/w) was determined mathematically by multiplying total nitrogen content by the general factor for cereal protein determination, 6.25, as described in the AOAC Official Method 992.23 (AOAC Association of Official Analytical Chemists, 1995).

### Nitrate and nitrite reductase and nitrogenase activity

Nitrate and nitrite reductase activities in the nodules were assayed using the methods of Hageman and Hucklesby (1971) and Finka et al. (1977), respectively. Phosphate buffer (pH 7.5) containing 0.1 M potassium nitrate and 5% *n*-propanol was used to extract the reductases. The optical density of the samples was measured spectrophotometrically at 550 nm. A standard curve of potassium nitrite was used as a reference. The activity of nitrate and nitrite reductases was expressed as μmNO_2_ released/h/g FW and μmNO_2_ disappeared/h/g fresh wt, respectively. Nitrogenase (EC 1.7.99.2) activity (ARA) was determined by acetylene reduction with the known weight of the nodules collected from all nodulated root portions of the plants, following the method of Herdina and Silsbury (1990). Gas samples were analyzed for ethylene produced in the reaction using a Shimadzu GC-14B gas chromatograph equipped with a Porapak R column (Ligero et al., 1986).

### Determination of photosynthetic pigments

Photosynthetic pigments were extracted from leaf samples in 80% acetone as described by Arnon (1949). Fresh leaf samples (0.5 g) were extracted in 80% acetone (v/v). The extracted material was centrifuged at 10, 000 × g for 10 min. The optical density of the supernatants was recorded at 480, 645, and 663 nm using a UV-visible spectrophotometer (T80 UV/VIS spectrometer, PG Instruments Ltd., USA). A blank with 80% acetone served as the control.

### Estimation of acid and alkaline phosphatase activity

Fresh root samples were extracted with 0.1 M acetate buffer (pH 5.0) and 0.1 M Tris HCI buffer (pH 8.2) for acid phosphatase (AP) (EC 3.1.3.2) and alkaline phosphatase (ALP) (EC 3.1.3.1), respectively, as described by Gianinazzi-Pearson and Gianinazzi (1976). AP and ALP were assayed following the methods of Ikawa et al. (1964) and Torriani (1967), respectively. In these methods, the reaction mixture of AP contained 0.2 ml of enzyme extract and 1.0 ml of 5.5 mM *p*-nitrophenol phosphate in 5.5 mM citrate buffer (pH 4.8). For the ALP assays, 0.05 M Tris-citrate (pH 8.5) was used instead of citrate buffer (pH 4.8). The reaction mixtures were incubated at 37°C for 30 min, and the reactions were stopped by adding 10 ml of 200 mM NaOH. Absorbance was recorded at 410 nm, and activity was expressed as μmol *p*-nitrophenol released min/mg protein.

### Determination of mineral contents

To determine the mineral contents, oven-dried leaves were powdered, and the powder was digested with 98% H_2_SO_4_ and 30% H_2_O_2_. The total nitrogen (N) content in leaf tissues was determined following the semi-micro Kjedahl procedure using a nitrogen analyzer (Kjedahl 2300; FOSS, Hoganas, Sweden). The phosphorus (P) content was estimated using the vanadomolybdophosphoric colorimetric method (Jackson, 1962). A standard curve (10–100 μg/ml) of potassium dihydrogen phosphate (KH_2_PO_4_) was used as the reference. The contents of Na^+^, K^+^, Mg^2+^, and Ca^2+^ in plant leaves were estimated as described by Wolf (1982) using a flame photometer (Jenway Flame Photometer, Bibby Scientific Ltd., Stone-Staffs, UK). The chloride (Cl^−^) concentration was directly estimated in the digested extracts using a chloride analyzer (Model 926, Sherwood Scientific Ltd., Cambridge, UK), as described by Abd_Allah et al. (2015b).

### Statistical analyses

Duncan's multiple range test was performed using one-way analysis of variance (ANOVA) for a completely randomized design by SPSS-21 software, and significant differences in means were determined by the least significant differences (LSD) (*p* = 0.05) test. In addition, the correlation coefficients among the parameters studied were calculated and are presented in Tables 3, 5, Table S3.

## Results

### Identification of microorganisms

Using nucleotide homology and phylogenetic analysis of the 16S rRNA gene sequences, we grouped the microbe that showed the highest salt tolerance in a cluster containing *B. subtilis* (GenBank Accession Number: JX188065.1) with 99% sequence similarity. The genome sequence of *B. subtilis* BERA71 was deposited in GenBank with the accession number KX090253. The strain produced IAA in nutrient broth containing 1.5% (260 mM) NaCl and cellulose and also solubilized mineral phosphorus from tricalcium phosphate used in solid medium.

**Figure 1:**
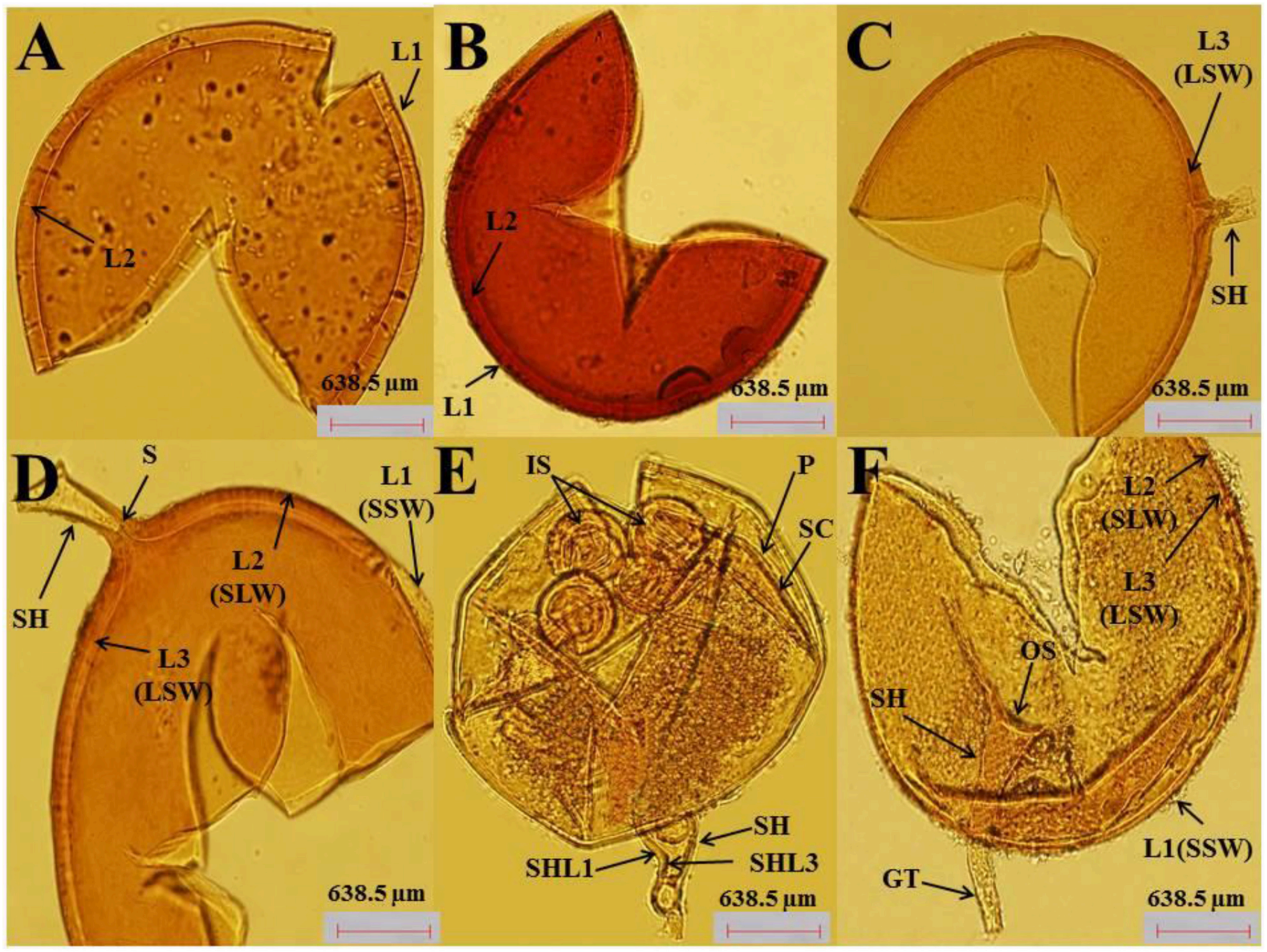
**(A–F)** (40X). AMF spores were isolated from soil samples of *A. gerrardii*. **(A,B)** The microscopic investigation indicated that the crushed spores of *C. etunicatum* were globose, sub-globose in shape, orange to red-brown in color and consisted of two layers (L1 and L2),. **(C,D)** Describes the spore morphology of R. intraradices as globose to subglobose in shape and whose crushed spores consisted of three layers (L1, L2, and L3) and prominent subtending hyphae. **(E,F)** The microscopic investigation revealed that the spores of *F. mosseae* are clustered together within a compact peridium. The shape of the spores was globose to subglobose, and the spore wall consisted of three layers (L1, L2, and L3).

Photomicrographs in (Figures 1A–F) showing spore morphology of AMF (*Claroideoglomus etunicatum, Rhizophagus intraradices, Funneliformis mosseae*) used in the current study. Figures 1A,B Crushed spore of *C. etunicatum* showing depressions on the surface and two wall layers: L1, an outer permanent rigid layer with some plasticity and an uneven outer surface. L2, A layer consisting of laminae that increase in thickness (~8.0 μm thick) is rigid, exhibiting some swelling and spreading when broken. *R. intraradices*: Figures 1C,D Crushed yellow-brown spore, globose or subglobose in shape, consists of three layers. L1, Outer layer has a sloughing spore wall (SSW), hyaline, mucilaginous spores that stain pale pink. L2: A rigid hyaline layer attached firmly to the underlying laminae [L3] as a sub-laminae layer of the spore wall (SLW). L3: Inner layer as laminae of the spore wall (LSW), which is continuous with the innermost layer of the subtending hypha. The subtending hypha (SH) is cylindrical to slightly flared with three layers that are continuous with the three layers of the spore wall. The plug partitions the spore from the hyphal contents (septum, S). A septum (S) occludes the hyphal attachment of a thin-walled spore of the pale morph close to the spore base*. F. mosseae*: Figures 1E,F Crushed spore showing the wall structure as three layers. L1, Outer wall, hyaline, mucilaginous, forming a sloughing granular layer (SSW). L2, Hyaline, generally rigid, consisting of a thin adherent sublayer attached firmly to the underlying laminae (L3). L3, Inner layer consisting of laminae with minute depressions covering the surface and separating L1 and L2. The sporocarp (SC) is subglobose, light brown, and surrounded by a peridium (P). Developed intact spores (IS) were observed. The subtending hypha is funnel-shaped with a width ranging between 16 and 32 μm (Figure 1F). As shown in Figure 1E, the subtending hypha consists of two layers (L1, L3), with the sum of thickness ranging between 2.4 and 4.8 μm. A germ tube emerges from the lumen (funnel-shaped) of the subtending hypha, originating from the occlusion-recovered septum. Sporocarps dramatically produce numerous infective hyphae (Figure 1E). The spores in the current isolates from soil adhering to the roots of *A. gerrardii* were identified as *F. mosseae* (syn. *Glomus mosseae*), *R. intraradices* (syn. *Glomus intraradices*), and *C. etunicatum* (syn. *Glomus etunicatum*).

### Plant growth parameters

The germination of *A. gerrardii* seeds was tested at NaCl concentrations of 50–300 mM. The results showed that increases in salt concentration decreased the germination rate of the seeds compared to that of the control seeds (water only) (from 87 to 3%). Because germination of the seeds was strongly impaired by 300 mM NaCl, we used 250 mM NaCl in all further salt stress experiments.

The growth of *A. gerrardii* was strongly impaired by salinity when plants were not inoculated with AMF or endophytic bacteria. Salinity reduced shoot height and shoot dry weight by 61 and 62%, respectively, and root length and dry weight were reduced by 35 and 38%, respectively (Table 1), compared with non-stressed plants. Salt-stressed shoots were 35% shorter and root depth 61% less in plants treated with 250 mM NaCl than non-stressed plant shoots and roots.

**Table 1:** **Shoot and root growth of *Acacia gerrardii* under salt stress after inoculation with *B. subtilis* and AMF alone and in combination**.

**Treatments**		**SH (cm)**	**SDW (g)**	**RD (cm)**	**RDW (g)**	**SH/RD**	**SDW/RDW**
0 mM NaCl	Control	53.4 ± 1.7	1.94 ± 0.2	70.9 ± 2.1	2.78 ± 0.3	0.75 ± 0.04	0.70 ± 0.03
	BS	78.4 ± 1.9	2.95 ± 0.3	87.0 ± 2.4	3.66 ± 0.4	0.90 ± 0.05	0.80 ± 0.04
	AMF	61.4 ± 1.5	2.51 ± 0.2	75.7 ± 2.3	3.24 ± 0.5	0.81 ± 0.06	0.77 ± 0.03
	BS + AMF	80.2 ± 2.1	3.55 ± 0.4	92.1 ± 3.1	3.87 ± 0.4	0.87 ± 0.06	0.91 ± 0.05
250 mM NaCl	Control	20.8 ± 1.0	0.74 ± 0.1	46.0 ± 1.2	1.72 ± 0.1	0.45 ± 0.02	0.43 ± 0.01
	BS	41.6 ± 1.4	1.61 ± 0.2	63.6 ± 1.5	2.42 ± 0.2	0.65 ± 0.03	0.67 ± 0.03
	AMF	29.1 ± 1.2	1.13 ± 0.1	56.8 ± 1.3	2.09 ± 0.2	0.51 ± 0.03	0.54 ± 0.03
	BS + AMF	48.1 ± 1.3	1.82 ± 0.2	66.4 ± 1.4	2.63 ± 0.1	0.72 ± 0.04	0.69 ± 0.04
	LSD *p* < 0.05:	2.80	0.08	1.31	0.12	0.04	0.04

*SH, shoot height; SDW, shoot dry weight; RD, root depth; RDW, root dry weight; ±, standard deviation*.

Plant growth depended strongly on the presence or absence of both AMF and the endophytic bacterium, BS. The inoculation of plants with *B. subtilis* alone or with AMF enhanced the root and shoot growth of non-stressed *A. gerrardii*, and the difference between the uninoculated and co-inoculated plants was significant (Table 1). Roots inoculated with the endophytic BS alone were significantly longer (22%) than uninoculated roots and were longer (14%) than roots inoculated with AMF alone (Table 1). In general, plant growth responded positively to the BS inoculation compared to AMF alone.

Salt-stressed *A. gerrardii* inoculated with endophytic *B. subtilis* alone grew better than salt-treated uninoculated plants. Root dry weight increased by 40% and shoot dry weight by 118% in the presence of 250 mM NaCl (Table 1). Shoot height and root length also responded positively to a single inoculation of BS. Root and shoot growth varied between treatments with the single inoculants and were higher in plants inoculated with the endophytic BS strain than with AMF. However, the interaction of AMF and rhizobia affected plant productivity positively compared to a single inoculation. Co-inoculated *A. gerrardii* had significantly higher shoot and root weight than plants inoculated with AMF or BS alone under NaCl stress (Table 1, Figure S1).

### Colonization of AMF in plant roots

**Figure 2:**
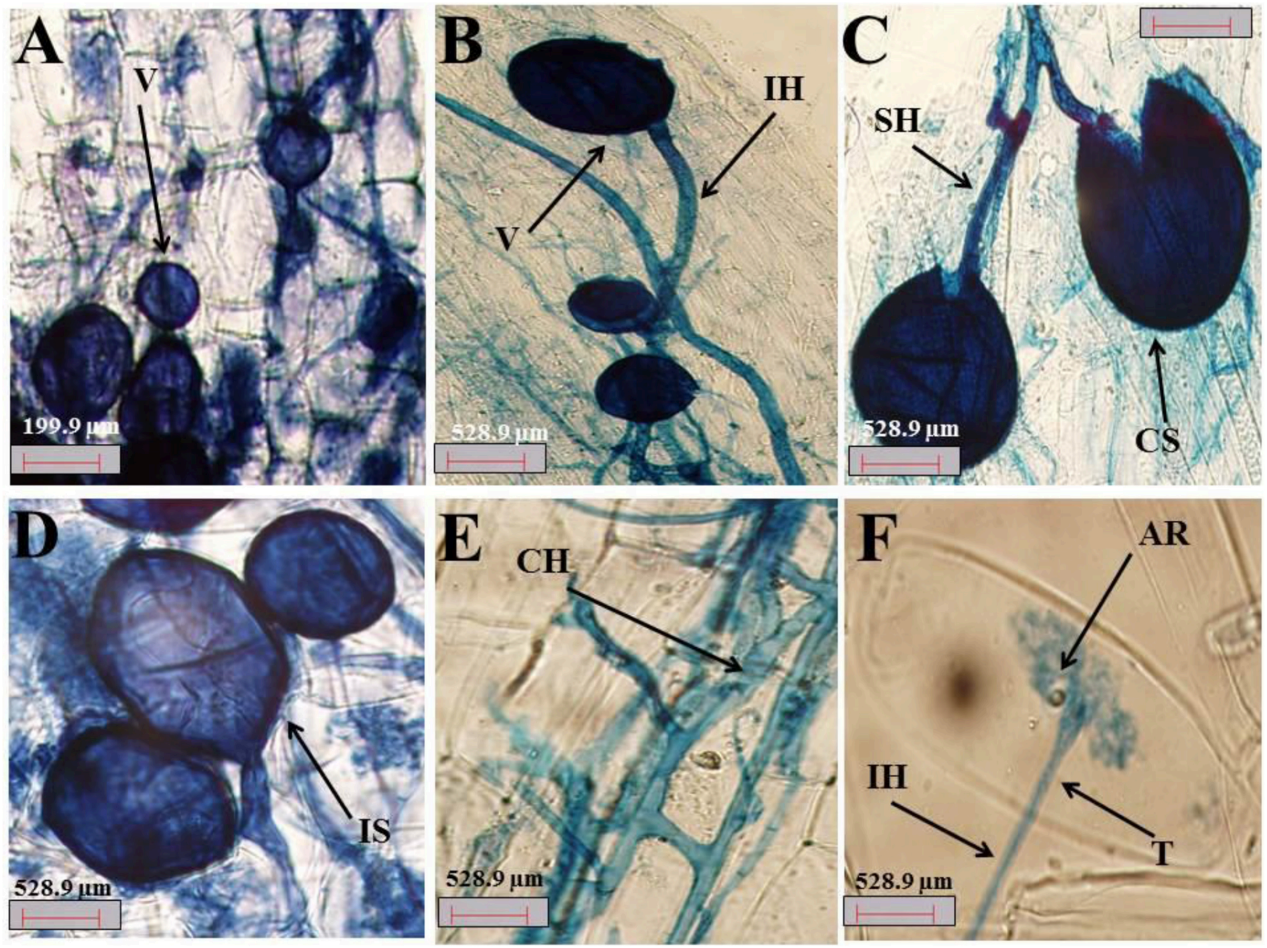
**(A–F)** Photomicrographs of structural colonization of AMF in the roots of *A. gerrardii*. **(A)** Vesicles (V); **(B)** intraradical hypha (IH), and vesicles (V). **(C)** crushed spore (CS) and subtending hypha (SH). **(D)** Intact spore (IS). **(E)** Coiled hyphae (CH). **(F)** Arbuscule (AR), trunk (T) and Intraradical hypha (IH).

The colonization of fine *A*. *gerrardii* roots by AMF is shown in Figures 2A–F. The roots were colonized with different AMF morphological structures, such as vesicles (Figure 2A), spores (Figure 2C), mycelium hyphae, intraradical hyphae (Figure 2B), subtending hyphae (Figure 2C), coiled hyphae (Figure 2E), and arbuscules (Figure 2F).

The percentage of 67.6, 55.8, and 13.8 of AMF that colonized the roots of *A*. *gerrardii* were in the form of mycelia, vesicles and arbuscules, respectively, with a total spore density of 707.8 spores/g of experimental soil (Table S1). NaCl stress decreased spore density, the presence of mycelia, vesicles and arbuscules by 24.8, 63.6, 20.7, and 60.4%, respectively, compared with the control treatment. Endophytic BS alleviated the adverse impacts of salt on spore density and mycorrhizal fungal colonization, and total spore count, mycelium, vesicles and arbuscules were increased by 27, 96, 14, and 23%, respectively, compared with those in the salt stress treatment group. In the absence of salt stress, the endophytic BS significantly increased both spore intensity and mycelium by 78.6 and 29.5%, respectively. However, both vesicles and arbuscules decreased by 48.38 and 44.92%, respectively, compared to those of the control treatment.

### Number of nodules, nodule fresh weight, and leghemoglobin content

The number of nodules, nodule FW and leghemoglobin content were reduced by 80.8, 80.04, and 80.6%, respectively, in salt-stressed plants relative to control, unstressed plants (Table 2). *A. gerrardii* grown in AMF-infected soil showed a higher number of nodules, nodule FW and leghemoglobin content (25.9, 51.8, and 18.02%, respectively) than plants grown in control soil in both non-saline and saline conditions. The root nodulation of *A. gerrardii* depended strongly on the presence or absence of AMF and/or the endophytic bacterium, *B. subtilis* (Table 2). BS and AMF alone improved the symbiotic performance of salt-stressed *A. gerrardii*. However, plants co-inoculated with AMF and *B. subtilis* produced three times more nodules, nodule FW, and leghemoglobin content in 250 mM NaCl than those in uninoculated plants.

**Table 2:** **Nodule number, fresh weight, leghemoglobin content, nitrate and nitrite reductase, and nitrogenase activity of *Acacia gerrardii* nodules under salt stress after inoculation with *B. subtilis* and AMF alone and in combination**.

**Treatments**		**NN**	**NFW**	**LG**	**NR**	**NiR**	**NG**	**CP**
0 mM NaCl	Control	57.1 ± 1.8	1.2 ± 0.04	6.2 ± 0.5	8.5 ± 0.7	6.1 ± 0.5	125.3 ± 10.3	593.1 ± 43.7
	BS	92.9 ± 2.3	2.1 ± 0.07	8.9 ± 0.7	11.1 ± 0.9	8.1 ± 0.7	175.5 ± 12.6	887.9 ± 62.1
	AMF	71.9 ± 1.9	1.8 ± 0.05	7.4 ± 0.7	9.8 ± 0.8	6.7 ± 0.5	151.3 ± 11.2	729.7 ± 51.7
	BS + AMF	106.4 ± 2.7	2.4 ± 0.09	9.1 ± 0.8	12.1 ± 1.2	8.8 ± 0.8	185.7 ± 13.4	997.5 ± 83.6
250 mM NaCl	Control	10.9 ± 0.4	0.2 ± 0.01	1.2 ± 0.1	5.2 ± 0.3	3.2 ± 0.2	34.0 ± 3.1	163.8 ± 13.7
	BS	45.9 ± 1.2	0.9 ± 0.02	4.7 ± 0.2	7.8 ± 0.5	4.3 ± 0.3	90.2 ± 8.4	493.5 ± 32.4
	AMF	31.4 ± 0.7	0.7 ± 0.01	3.1 ± 0.1	6.3 ± 0.4	4.1 ± 0.3	75.7 ± 6.4	408.1 ± 27.5
	BS + AMF	50.6 ± 1.6	1.0 ± 0.03	4.9 ± 0.3	8.1 ± 0.7	5.0 ± 0.4	98.7 ± 8.6	550.9 ± 37.8
	LSD *p* < 0.05	2.75	0.07	0.44	0.19	0.59	2.00	14.97

*NN, number of nodules; LG, leghemoglobin (mg g^−1^ nodule FW); NR, nitrate reductase (μm NO_2_ released/h/g FW), NiR, nitrite reductase (μm NO_2_ disappeared/h/g FW); NG, nitrogenase (μmole ethylene/mg nodule FW/h); CP, crude protein (total nitrogen content as mg/g dry weight × 6.25); ±, standard deviation*.

### Nitrate reductase, nitrite reductase, nitrogenase, and crude protein

Both AMF and endophytic BS applied alone to non-stressed and stressed plants increased the nitrate (NR) and nitrite reductase (NIR) as well as nitrogenase activity. Inoculation of AMF increased nitrate reductase, nitrite reductase and nitrogenase activity by 15.4, 10.1, and 20.7%, respectively, while BS enhanced the activity of these enzymes by 31.5, 32.2, and 40.06%, respectively (Table 2).

The combined inoculation of AMF and BS increased nitrate reductase, nitrite reductase, and nitrogenase activity in the nodules of *A. gerrardii* by 42.5, 43.7, and 48.2%, respectively (Table 2), while salt-stressed plants showed a 38.5, 46.4, and 72.8% decline in nitrate reductase, nitrite reductase, and nitrogenase activity, respectively. The differences in enzyme activities between uninoculated plants and those co-inoculated with AMF and BS under salt stress were significant, and nitrate reductase activity increased by 56%, nitrite reductase activity by 53% and nitrogenase activity by 189% (Table 2). The crude protein content increased in response to all microbial inoculation treatments regardless of whether salt was present. The combination of BS and AMF produced even better results because co-inoculated salt-stressed plant nodules contained significantly more protein than uninoculated salt-stressed ones (Table 2).

Table 3 show the correlations between salt, mycorrhiza, and bacteria with number of nodules, nodule FW, leghemoglobin nodule FW, nitrate reductase, nitrite reductase, nitrogenase, total nitrogen content, and crude protein. The results indicated a slight correlation between mycorrhiza and nitrite reductase (0.185). However, there was a strong correlation between the number of nodules and nitrate reductase (0.996).

**Table 3:** **Correlations (r) between salt, mycorrhiza, and bacteria with number of nodules, nodule fresh weight, leghemoglobin nodule fresh weight, nitrate reductase, nitrite reductase, nitrogenase, total nitrogen content, and crude protein**.

	**Sal**	**M**	**B**	**NN**	**NFW**	**LG**	**NR**	**NiR**	**NG**	**TN**	**CP**
Sal	1.00000	0.00000	0.00000	−0.80434	−0.82441	−0.84506	−0.80299	−0.84272	−0.86851	−0.79585	−0.79585
M		1.00000	0.00000	0.22748	0.27535	0.16619	0.21210	0.18576	0.22121	0.27398	0.27398
B			1.00000	0.52874	0.45420	0.45913	0.54203	0.38903	0.41955	0.51745	0.51745
NN				1.00000	0.98822	0.97559	0.99642	0.94935	0.98308	0.99350	0.99350
NFW					1.00000	0.96027	0.98864	0.93981	0.98060	0.98396	0.98396
LG						1.00000	0.97490	0.96162	0.98319	0.97358	0.97358
NR							1.00000	0.94095	0.98127	0.98920	0.98920
NiR								1.00000	0.94270	0.93546	0.93546
NG									1.00000	0.98790	0.98790
TN										1.00000	1.00000
CP											1.00000

*Sal, salt; M, mycorrhiza; B, Bacillus subtilis; NN, number of nodules; NFW, nodule fresh weight; LG, leghemoglobin (mg g^−1^ nodule FW); NR, nitrate reductase (μm NO_2_ released/h/g FW); NiR, nitrite reductase (μm NO_2_ disappeared/h/g FW); NG, nitrogenase (μmole ethylene/mg nodule FW/h); TN, total nitrogen content (mg/ g dry weight); CP, crude protein (total nitrogen content as mg/g dry weight × 6.25)*.

### Photosynthetic pigments

The photosynthetic pigments [chlorophyll *a* (ChlA), chlorophyll *b* (ChlB)], carotenoids and total chlorophyll (TChl) content in *A. gerrardii* were lower in plants without microbial inoculants or with only one inoculant. When plants were grown in the presence of AMF or BS, photosynthetic pigments increased, ChlA by 19.4 or 30.3%, ChlB by 12.6 or 28.3%, carotenoids by 41.8 or 62.5% and total chlorophyll content by 15.4 or 32.5%, respectively, compared to uninoculated plants (Table S2). Salinity reduced the content of ChlA, ChlB, carotenoids and TChl in *A. gerrardii* by 32.9, 40.5, 93.01, and 41.1%, respectively. Plants inoculated with endophytic BS and grown in soil infested with AMF showed an increase in the content of photosynthetic pigments. The content of ChlA, ChlB, carotenoids and TChl varied between the single inoculant treatments and was higher in plants inoculated with the endophytic BS strain than in those inoculated with AMF (Table S2). A weak correlation was found between mycorrhiza and carotenoids (0.203), while the strongest correlation (0.998) was between chlorophyll *a* + chlorophyll *b* and total photosynthetic pigments followed by the correlation between chlorophyll *a* and total photosynthetic pigments (0.992; Table S3).

### Nutrient contents

The nutrient contents decreased following treatment with 250 mM NaCl, N content by 72% and P by 59%, K by 53%, Mg by 65%, and Ca by 60% (Table 4). NaCl stress increased Na^+^ and Cl^−^ content compared to plants under non-stressed conditions. Generally, both inoculation treatments, either BS alone or combined with AMF, enhanced Na^+^, P, K^+^, Mg^2+^, and Ca^2+^ contents in non-stressed and salt-stressed *A. gerrardii* tissues (Table 4). However, the highest concentrations were detected in co-inoculated plant tissues grown in the presence of 250 mM NaCl. The correlations between salt, mycorrhiza, and bacteria with sodium, potassium, magnesium, calcium and chloride contents are shown in Table 5. The strongest correlation was between calcium and potassium (0.935), while the weakest correlation was between mycorrhiza and potassium (0.211).

**Table 4:** **Mineral contents of *Acacia gerrardii* under salt stress after inoculation with *B. subtilis* and AMF alone and in combination**.

**Treatments**		**Accumulation of elements (mg/g dry weight)**						
	**Na**	**K**	**Mg**	**Ca**	**Cl**	**N**	**P**
0 mM NaCl	Control	11.3 ± 0.9	28.3 ± 2.5	2.1 ± 0.2	2.8 ± 0.2	8.1 ± 0.6	94.8 ± 8.2	1.6 ± 0.1
	BS	15.7 ± 1.2	32.9 ± 3.1	3.5 ± 0.3	4.0 ± 0.3	5.2 ± 0.3	142.0 ± 12.4	1.8 ± 0.1
	AMF	12.4 ± 1.1	29.6 ± 2.7	2.2 ± 0.2	3.6 ± 0.3	7.0 ± 0.5	116.7 ± 10.3	2.9 ± 0.2
	BS + AMF	17.0 ± 1.4	35.1 ± 3.2	2.8 ± 0.2	4.6 ± 0.4	4.6 ± 0.2	159.6 ± 13.5	3.2 ± 0.2
250 mM NaCl	Control	37.3 ± 3.1	13.3 ± 1.1	0.7 ± 0.04	1.1 ± 0.06	27.2 ± 1.9	26.2 ± 2.3	0.6 ± 0.04
	BS	26.2 ± 2.4	23.5 ± 2.1	1.6 ± 0.1	2.2 ± 0.2	13.7 ± 1.1	78.9 ± 6.4	1.0 ± 0.07
	AMF	28.8 ± 2.5	18.3 ± 1.6	1.0 ± 0.08	2.0 ± 0.2	19.4 ± 1.7	65.3 ± 6.1	1.2 ± 0.1
	BS + AMF	25.0 ± 2.3	26.6 ± 2.4	1.8 ± 0.1	2.4 ± 0.2	11.0 ± 0.8	88.1 ± 7.6	1.4 ± 0.1
	LSD *p* < 0.05	1.51	0.54	0.11	0.25	0.28	2.14	0.16

*Na, sodium; K, potassium; Mg, magnesium; Ca, calcium; N, nitrogen; P, phosphorous; ±, standard deviation*.

**Table 5:** **Correlations (r) between salt, mycorrhiza, and bacteria with sodium, potassium, magnesium, calcium, and chloride**.

	**Sal**	**M**	**B**	**Na**	**K**	**Mg**	**Ca**	**Cl**
Sal	1.00000	0.00000	0.00000	0.88260	−0.80382	−0.79170	−0.81818	0.79031
M		1.00000	0.00000	−0.10702	0.21197	−0.02390	0.28581	−0.20716
B			1.00000	−0.08547	0.51986	0.54310	0.42853	−0.46092
Na				1.00000	−0.85107	−0.75276	−0.77220	0.90348
K					1.00000	0.91692	0.93540	−0.97884
Mg						1.00000	0.90114	−0.88184
Ca							1.00000	−0.88885
Cl								1.00000

*Sal, salt; M, mycorrhiza; B, Bacillus subtilis; Na, sodium; K, potassium; Mg, magnesium; Ca, calcium; Cl, chloride*.

### Acid and alkaline phosphatases

**Figure 3:**
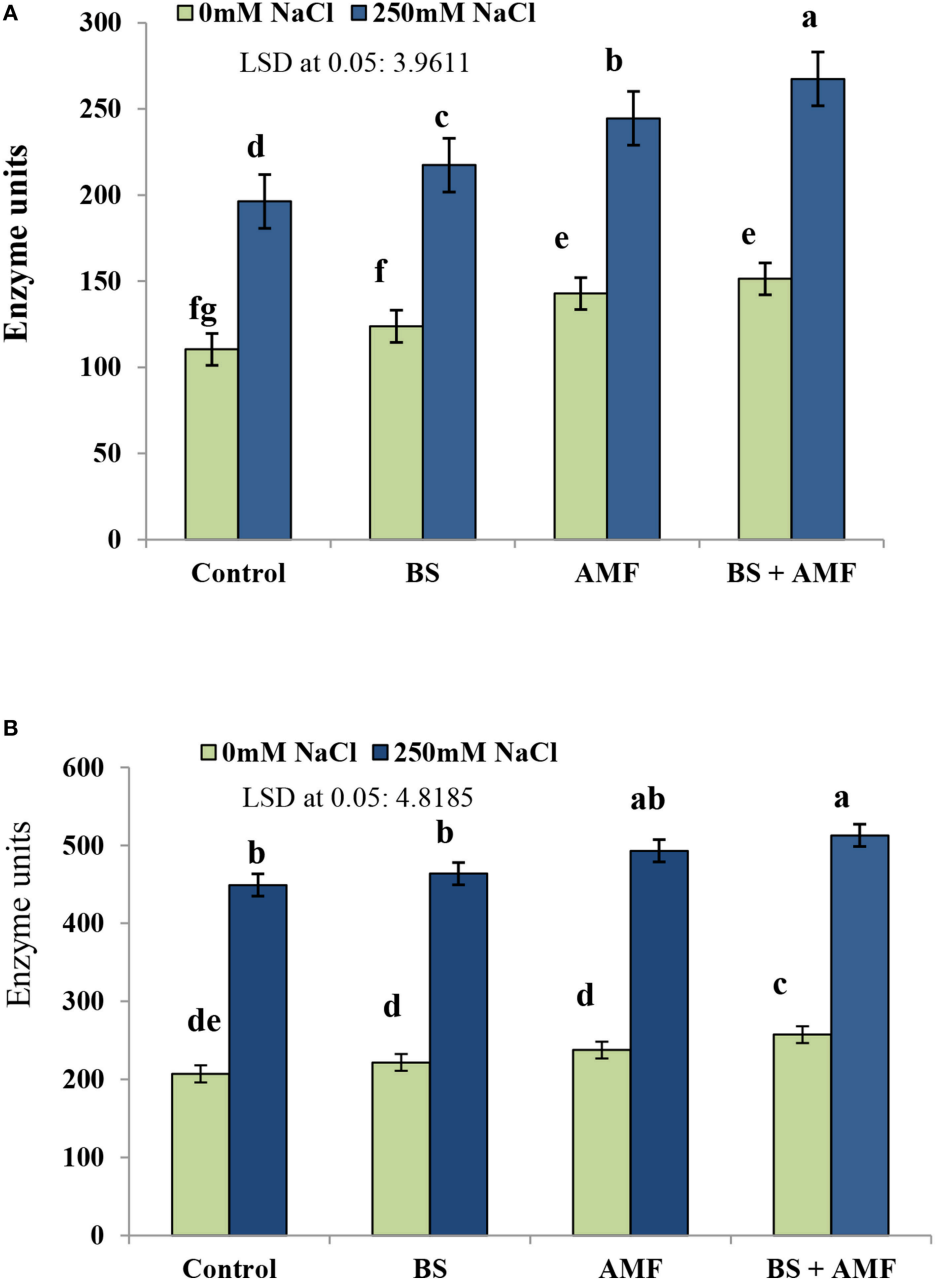
**The enzymes activity (Unit) of acid phosphatase (A) and alkaline phosphatase (B) in fresh root of *Acacia gerrardii* in response to endophytic *B. subtilis* under salt stress (250 mM NaCl)**. Columns represent means for five plants (*N* = 5). Error bars show standard error. Columns with different letters indicate significant differences between treatments at *P* < 0.05.

Acid phosphatase (AP) activity significantly increased as a consequence of salinity in uninoculated plant tissues (Figure 3). The acid and ALP increased by 78.3 and 116.9%, respectively, in response to 250 mM NaCl compared to non-stressed control plants. Inoculation with AMF had a stimulatory effect on the activity of both phosphatases in non-stressed and stressed plants (Figures 3A,B). Following combined inoculation of AMF and BS, AP activity increased by 142.7 and 147.3% in 250 mM NaCl and in unstressed control plants, respectively.

## Discussion

The results from our experiments showed that a tripartite interaction of AMF, endophytic BS and the host plant *A. gerrardii* may have the potential to remediate degraded sites and can compensate for abiotic stresses due to climate change. Although, several reports have shown a positive effect of dual inoculation with AMF and PGPR on plant growth and stress tolerance, such as AMF with *Enterobacter radicincitans* on faba bean (*Vicia faba*) (Almethyeb et al., 2013), AMF with *Pseudomonas mendocina* on lettuce (*Lactuca sativa* L.) (Kohler et al., 2009), AMF with *Pseudomonas fluorescens* on common bean (*Phaseolis vulgaris* L.) (Neeraj and Singh, 2011), and AMF with *Azospirillum* on rice (*Oryza sativa*. L) (Ruíz-Sánchez et al., 2011), little is known about the interactions with endophytes as well as the underlying mechanisms. In this study, we observed a significant growth benefit of the synergistic association of *A. gerrardii* with AMF and endophytic *B. subtilis* under salt stress.

We found that salt stress reduced AMF colonization in *A. gerrardii*, consistent with observations made by Alqarawi et al. (2014a) in *Ephedra aphylla* and Hashem et al. (2015) in *Vigna unguiculata*. Our results showed that combined inoculation of plants with AMF and endophytic *B. subtilis* resulted in increased AMF colonization, which is an important indicator of plant nutrition. The synergistic interaction of *B. subtilis* and AMF altered *A. gerrardii* plant fitness under salt stress, significantly increasing plant biomass, nodulation, leghemoglobin, and crude protein content compared with untreated plants. An increase in plant growth and amelioration of salt stress by AMF was reported by Abd_Allah et al. (2015a) for sunflower (*Helianthus annuus* L.), by Aroca et al. (2013) for lettuce, and by Gomez-Bellot et al. (2015) for laurustinus plants (*Viburnum tinus* L.). Bacterial endophytes have also been shown to increase plant growth and tolerance to abiotic stresses, e.g., *P. fluorescens* (Ali et al., 2014), *Paenibacillus yonginensis* (Sukweenadhi et al., 2015), and *Bacillus* sp. (Andreolli et al., 2016). When colonizing plant tissues, microbes contribute multiple benefits, such as improved nutrient acquisition, tolerance to biotic and abiotic stresses, and modulation of plant defenses (Bordiec et al., 2011). In addition, the increase in nodulation may be due to a synergistic effect of the two types of microbes, namely symbiotic and endophytic, including naturally occurring rhizobia. Huang et al. (2011) reported a mutualistic symbiotic relationship between *B. subtilis* and a leguminous plant, *Robinia pseudoacacia* L. In this study, *B. subtilis* colonized plant roots in a manner similar to the infection of root hairs by rhizobia and formed bacteroids inside plant cortical cells. Inoculation of plants with cellulose-producing *B. subtilis* resulted in more nodules and higher nitrogenase activity than the uninoculated control and AMF-inoculated plants. Rhizobial symbionts penetrate deeper plant tissues by producing cellulase, which can completely erode the root-hair wall at the site of infection (Sindhu and Dadarwal, 2001). The synthesis of cell wall-degrading enzymes by *B. subtilis* could help explain the mechanism underlying rhizobial entry into target root hair cells to form nodules.

Increased nitrogenase activity following treatments with *B. subtilis* or *B. subtilis* combined with AMF resulted from their positive impact on the activity of enzymes such as nitrate reductase and nitrite reductase. Nitrate and nitrite reductase control the conversion of nitrate into ammonia and result in the formation of amino acids (Iqbal et al., 2015). In *Trifolium alexandrinum* L. and *Trifolium resupinatum* L. (Zarea et al., 2011), as well as in *Vicia faba* (Hashem et al., 2014), inoculation of AMF enhanced plant growth by improving nitrogenase activity and nodule formation.

High salt concentrations induce alterations in the synthesis of chlorophyll-related proteins and components of the oxygen-evolving complex, resulting in reduced photosynthetic efficiency (Alqarawi et al., 2014a,b). Altered *de novo* synthesis of proteins and the associated pigment-related components due to salinity has negative effects on the synthesis of photoassimilates and hence reduces the growth rate of plants. The combined inoculation of AMF and *B. subtilis* increase photosynthetic pigments, which may be a collective result of many positive changes induced by AMF and PGPR. The enhanced chlorophyll content due to AMF inoculation under normal as well as NaCl-stressed conditions corroborates the reports of Aroca et al. (2013) in lettuce, Alqarawi et al. (2014a) in *E. aphylla* and Abd_Allah et al. (2015a) in *Sesbania sesban*. Recently, in salt-stressed *Brassica juncea*, Ahmad et al. (2015) demonstrated the positive impact of *Trichoderma harzianum* inoculation on growth via improved chlorophyll synthesis. In *Ocimum basilicum* grown under water stress, inoculation of PGPR (*Pseudomonas* sp. and *Bacillus lentus*) increased chlorophyll synthesis as well photosynthetic electron transport and also mitigated the negative impact of water stress (Heidari and Golpayegani, 2012).

Salt stress inhibits the uptake of essential mineral elements, such as K, Mg, Ca, N, and P, because of the antagonistic relationship of sodium. By reducing the uptake of magnesium, salt stress affects plant photosynthetic efficiency by altering the synthesis of chlorophyll molecules. Reduced uptake of nitrogen directly affects the nitrogen metabolic potential as well as amino acid synthesis in plants (Näsholm et al., 2009). Improved plant nutrient uptake under salt stress conditions by AMF was reported in many studies e.g., for common bean (*Phaseolus vulgaris*) (Abd_Allah et al., 2015b), olive (*Olea europaea* L.) (Porras-Soriano et al., 2009), and wheat (*Triticum aestivum* L.) (Talaat and Shawky, 2014). Endophytic bacterial strains can also increase plant tolerance to abiotic stresses and improve nutrient uptake under multiple adverse conditions (Malfanova et al., 2011; Berg et al., 2013). The combined inoculation of plants with AMF and PGPR reduced the Na and Cl concentrations in plant tissues, thereby protecting salt-stressed plants from ionic and osmotic stress-induced changes. Their synergistic interaction resulted in an increase of N, P, and K uptake by plants. Endophytic bacterial strains appear to have some plant growth-promoting activities, such as IAA production and solubilization of phosphate, which together or alone might explain the capacity of *B. subtilis* to improve plant growth and nutrient acquisition (Malfanova et al., 2011). Messele and Pant (2012) observed improved nodulation, yield and P uptake in chickpea (*Cicer arietinum*) by phosphate-solubilizing *Pseudomonas*. An increase in P availability to plants through the action of phosphate-solubilizing bacteria (PSB) has also been reported for green gram (*Vigna radiata* L. Wilczek) (Vikram and Hamzehzarghani, 2008) and wheat (*T. aestivum* L.) (Panhwar et al., 2011). In earlier studies, it was reported that salt and drought stresses inhibit the production of plant growth regulators in plant tissues (Debez et al., 2001). The additional supply of hormones by endophytes in plant tissue can stimulate the root system, thereby facilitating the absorption of more nutrients from the soil, especially under stress conditions (Malfanova et al., 2011; Berg et al., 2013). Studies by Kavino et al. (2010), Heidari et al. (2011), and Lavakush et al. (2014) also support the positive impact of PGPR on the mineral nutrient status of plants under normal and stressed conditions in determining the physiological strength of a plant. Improved Mg content affects chlorophyll, while P and N contribute to the energy budget of a cell, and Ca serves as an important cellular messenger for downstream signaling (Ahanger et al., 2014b). Improved K uptake is associated with reduced Na uptake in AMF- and PGPR-inoculated plants and results in an enhanced K/Na ratio, an important aspect for the maintenance of physiological cellular functioning (Abd_Allah et al., 2015a,b; Ahanger et al., 2015).

Phosphatases are responsible for the hydrolysis of a range of organic P compounds and provide mineral phosphate to the plant (Tazisong et al., 2015). In our study, increased acid and ALP was observed in *A. gerrardii* grown under salt stress. Similarly, enhanced AP activity under salt stress was also observed in *Medicago sativa* (Ehsanpour and Amini, 2003) and may be due to the fact that salt stress suppresses plant growth, P uptake, transport and utilization (Dracup et al., 1984). An increase in the activities of phosphatases (alkaline and acidic phosphatase) following the combined inoculation of *A. gerrardii* plants with AMF and *B. subtilis* support the findings of Amaya-Carpio et al. (2009) in *Ipomoea carnea* sp. *fistulosa* and Kebrabadi et al. (2014) in *Fraxinus rotundifolia*. During P deficiency or impaired P uptake, plants release or activate acid phosphates and exude carboxylates and phosphatases to enhance P solubilization and uptake (Veneklaas et al., 2003). An increase in the activity of phosphatases in inoculated plants, either with AMF or *B. subtilis*, alone or in combination, can contribute to the release of bound P to maximize its uptake and transport. Maize plants inoculated with *B. subtilis* showed an increase in phosphatase activity compared to uninoculated controls (Hussain et al., 2013). Salinity-stressed plants showed lower P accumulation in the above-ground plant parts, which may be due to the toxicity of high salt concentrations.

## Conclusion

Our observations in this study indicate that endophytic bacteria and AMF that live within the plant tissues of *A. gerrardii* are coordinately involved in the plant's adaptation to stress tolerance. Inoculation of plants with AMF and endophytic *B. subtilis* increased plant growth and nutrient acquisition and improved symbiotic performance of *A. gerrardii*. In addition, endophytic *B. subtilis* increased AMF germination and root colonization of *A. gerrardii* under salt stress.

## Author contributions

AAH, Provided the seeds and seedlings. AH, Provided AMF and mycological analysis. EA, All biochemical analysis, writing and graphing. AAA, Statistical analysis. SW, Revision and editing. DE, writing, revision and editing.

### Conflict of interest statement

The authors declare that the research was conducted in the absence of any commercial or financial relationships that could be construed as a potential conflict of interest.

## Footnotes

^1^INVAM - International Culture Collection of Arbuscular & Vesicular-Arbuscular Mycorrhizal Fungi. Available online at: www.invam.caf.wvu.edu/index.html (Accessed February 6, 2012).
