# Supplementary material for: The Interaction between Arbuscular Mycorrhizal Fungi and Endophytic Bacteria Enhances Plant Growth of Acacia gerrardii under Salt Stress
Source: Front Microbiol. 2016 Jul 19;7:1089. doi: 10.3389/fmicb.2016.01089 (PMC4949997; doi:10.3389/fmicb.2016.01089)
Supplement: Supplementary file 1 [file DataSheet1.docx]

Table S1. Spore density (SD) and number of mycelia (M), vesicles (V) and arbuscules (A) of mycorrhizal fungi (%) in the roots of *Acacia* *gerrardii* under salt stress in response to inoculation with endophytic *B. subtilis.*

| **Treatments** | | **SD*** | **M** | **V** | **A** |
| --- | --- | --- | --- | --- | --- |
| **0 mM NaCl** | AMF | 707.8±10.2 | 67.6±2.7 | 55.8±2.2 | 13.8±0.3 |
|  | BS+AMF | 1186.4±11.7 | 87.6±3.1 | 28.8±1.4 | 7.6±0.2 |
| **250 mM NaCl** | AMF | 531.6±8.4 | 24.6±1.2 | 44.2±1.8 | 15.6±0.4 |
|  | BS+AMF | 675.4±9.2 | 48.4±2.1 | 50.4±2.2 | 19.2±0.9 |
| LSD p<0.05 | | 31.09 | 11.52 | 13.08 | 11.14 |

*Spore density as number of spores/100 g soil; ±: standard deviation.

Table S2. Chlorophyll *a*, chlorophyll *b*, total chlorophylls and carotenoid contents of *Acacia gerrardii* under salt stress after inoculation with *B. subtilis* and AMF alone and in combination

|  | | **Photosynthetic pigments (mg/g FW)** | | | | | | | |
| --- | --- | --- | --- | --- | --- | --- | --- | --- | --- |
| **Treatments** | | **Chl *a*** | **Chl *b*** | | ***a*+*b*** | ***a*/*b*** | **CT** | **Total PP** | |
| **0 mM NaCl** | Control | 1.24±0.05 | 0.80±0.02 | | 2.05±0.12 | 1.55±0.04 | 0.20±0.01 | 2.25±0.14 |  |
|  | BS | 1.62±0.08 | 1.03±0.03 | | 2.65±0.18 | 1.58±0.04 | 0.33±0.02 | 2.99±0.18 |  |
|  | AMF | 1.40±0.06 | 0.90±0.02 | | 2.31±0.14 | 1.55±0.04 | 0.29±0.02 | 2.60±0.15 |  |
|  | BS + AMF | 1.76±0.09 | 1.09±0.04 | | 2.86±0.19 | 1.61±0.06 | 0.35±0.03 | 3.21±0.28 |  |
| **250 mM NaCl** | Control | 0.83±0.01 | 0.47±0.01 | | 1.31±0.07 | 1.75±0.07 | 0.12±0.01 | 1.32±0.06 |  |
|  | BS | 1.13±0.02 | 0.64±0.02 | | 1.77±0.09 | 1.75±0.07 | 0.10±0.01 | 1.87±0.07 |  |
|  | AMF | 1.02±0.02 | 0.60±0.02 | | 1.62±0.07 | 1.70±0.06 | 0.06±0.01 | 1.68±0.05 |  |
|  | BS + AMF | 1.21±0.03 | 0.75±0.03 | | 1.96±0.11 | 1.61±0.05 | 0.14±0.01 | 2.10±0.14 |  |
|  | LSD p<0.05 | 0.045 | 0.029 | 0.053 | | 0.084 | 0.015 | 0.05 | |

Chl *a*, chlorophyll *a*; Chl *b*, chlorophyll *b*; CT, carotenoids; total PP, total photosynthetic pigments (mg/g FW); FW, fresh weight; ±: standard deviation.

Table S3. Correlations (r) between salt, mycorrhiza, and bacteria with chlorophyll *a*, chlorophyll *b*, carotenoids and total photosynthetic pigments.

|  | Sal | M | B | Ch *a* | Ch *b* | Ch *a*+*b* | Ch *a*/*b* | Carot | TPP |
| --- | --- | --- | --- | --- | --- | --- | --- | --- | --- |
| Sal | 1.00000 | 0.00000 | 0.00000 | -0.79218 | -0.84092 | -0.81794 | 0.58336 | -0.89163 | -0.83605 |
| M |  | 1.00000 | 0.00000 | 0.24575 | 0.24426 | 0.24688 | -0.16837 | 0.20368 | 0.23940 |
| B |  |  | 1.00000 | 0.52326 | 0.45521 | 0.49885 | -0.00181 | 0.37272 | 0.47606 |
| Ch *a* |  |  |  | 1.00000 | 0.97100 | 0.99511 | -0.39035 | 0.95894 | 0.99226 |
| Ch *b* |  |  |  |  | 1.00000 | 0.98987 | -0.59092 | 0.97608 | 0.99142 |
| Ch *a*+*b* |  |  |  |  |  | 1.00000 | -0.47599 | 0.97282 | 0.99895 |
| Ch *a*/*b* |  |  |  |  |  |  | 1.00000 | -0.55500 | -0.49368 |
| Carot |  |  |  |  |  |  |  | 1.00000 | 0.98241 |
| TPP |  |  |  |  |  |  |  |  | 1.00000 |

Sal, salt; M, mycorrhiza; B, *Bacillus subtilis*; Chl *a*, chlorophyll *a*; Chl *b*, chlorophyll *b*; CT, carotenoids; TPP, total photosynthetic pigments (mg/g FW).


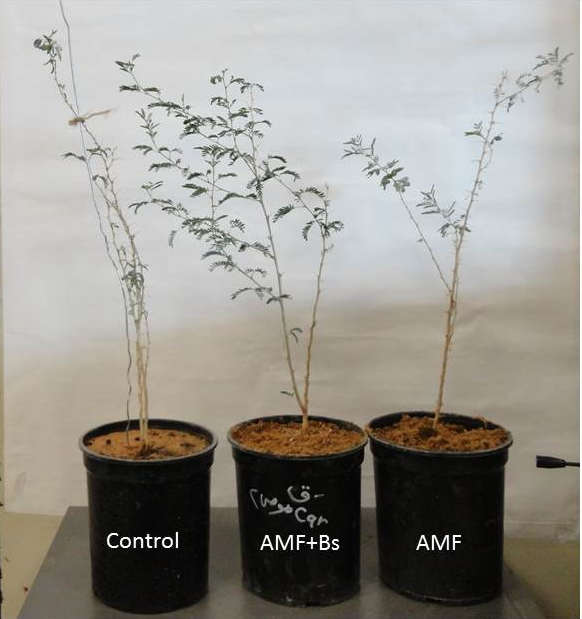


Figure S1. Plant growth of *A. gerrardii* inoculated in combination with *B. subtilis* and AMF; and with AMF alone under saline soil condition.
